# Robotic surgery for median arcuate ligament syndrome: technical aspects and imaging correlation

**DOI:** 10.1590/0102-67202026000004e1933

**Published:** 2026-06-12

**Authors:** Michele Andreza Fidelis SIQUEIRA, Rafael Hardman DAIM, Maria Eduarda Hardman KNAESEL, Ricardo Lemos COTTA-PEREIRA

**Affiliations:** 1 Instituto D’Or de Pesquisa e Ensino, Medical Residency Program in Digestive Surgery – Rio de Janeiro (RJ), Brazil.; 2 Instituto Técnico Educacional Souza Marques, Medical School – Rio de Janeiro (RJ), Brazil.

**Keywords:** Abdominal Pain, Median Arcuate Ligament Syndrome, Robotic Surgical Procedures, Celiac Artery, Dor Abdominal, Síndrome do Ligamento Arqueado Mediano, Procedimentos Cirúrgicos Robóticos, Artéria Celíaca

## Abstract

**Background:**

Median arcuate ligament syndrome (MALS) is an uncommon condition characterized by chronic and intermittent abdominal pain, typically postprandial, and weight loss, caused by extrinsic compression of the celiac trunk by the median arcuate ligament. The pathophysiology of the disease is not fully understood. Diagnosis is challenging due to nonspecific symptoms, and requires a careful correlation between clinical findings and imaging studies.

**Aims:**

To demonstrate the feasibility, safety, and technical aspects of robotic median arcuate ligament release using a case-based approach with detailed imaging correlation.

**Methods:**

The authors report the technical aspects in an elderly male patient with typical symptoms of MALS, who underwent robotic-assisted median arcuate ligament release using the da Vinci X platform. Preoperative evaluation included CT angiography and color Doppler ultrasound, demonstrating focal proximal celiac trunk stenosis.

**Results:**

The robotic approach allowed precise dissection and complete decompression of the celiac trunk without intraoperative complications. Postoperative imaging demonstrated resolution of the stenosis and normalization of Doppler flow parameters. The patient experienced complete symptom resolution, and remained asymptomatic after one year of follow-up.

**Conclusions:**

Robotic median arcuate ligament release is a safe and effective minimally invasive option, providing excellent visualization and precise dissection in a challenging anatomical region. This technique should be considered a valuable approach for selected patients with MALS.

## ARTICLE HIGHLIGHTS

Median arcuate ligament syndrome (MALS), also known as Dunbar syndrome, is an uncommon condition.

It is characterized by chronic abdominal pain resulting from external compression of the celiac trunk by the median arcuate ligament.

The treatment consists of surgical decompression of the celiac trunk.

Robotic median arcuate ligament release using the da Vinci platform is a safe, feasible, and effective minimally invasive approach for the treatment of MALS.

## Summary

**CENTRAL MESSAGE:**

Robotic surgery, due to the surgeon’s ergonomics and the stability of the arms and instruments, wrist movement, and magnified threedimensional vision, allows for facilitated and safe access to the origin of the celiac artery as well as the dissection of fibrous tissues, innervation, and lymphatic vessels surrounding it. Given this, robotic surgery becomes feasible, effective, and safe for treating this syndrome, and it should be considered a preferred option in selected patients.

**PERSPECTIVES:**

The median arcuate ligament syndrome should be considered in cases of abdominal pain with no defined causes. A high index of suspicion can diagnose and treat more cases. Moreover, it is expected that robotic surgery will become increasingly accessible, allowing for an expansion of its indications.

## VISUAL ABSTRACT

**Figure:**
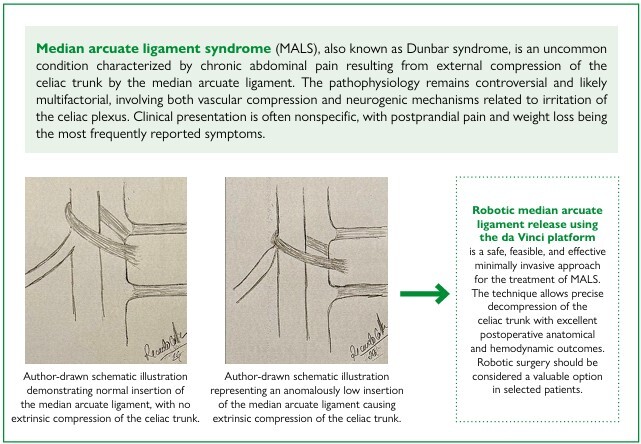

## RESUMO

**Racional:**

A síndrome do ligamento arqueado mediano (SLAM) é uma condição incomum caracterizada por dor abdominal crônica e intermitente, tipicamente pós-prandial, com perda de peso, causada pela compressão extrínseca do tronco celíaco pelo ligamento arqueado mediano. A fisiopatologia da doença não é totalmente compreendida. O diagnóstico é desafiador devido aos sintomas inespecíficos e requer uma correlação cuidadosa entre os achados clínicos e os exames de imagem.

**Objetivos:**

Demonstrar a viabilidade, segurança e aspectos técnicos da liberação robótica do ligamento arqueado mediano utilizando uma abordagem baseada em casos clínicos com correlação detalhada por imagem.

**Métodos:**

Os autores relatam os aspectos técniocos em um paciente idoso do sexo masculino com sintomas típicos de SLAM foi submetido à liberação robótica do ligamento arqueado mediano utilizando a plataforma da Vinci X. A avaliação pré-operatória incluiu angiotomografia computadorizada e a ultrassonografia Doppler colorida, demonstrando estenose focal proximal do tronco celíaco.

**Resultados:**

A abordagem robótica permitiu a dissecção precisa e a descompressão completa do tronco celíaco sem complicações intraoperatórias. Os exames de imagem pós-operatórios demonstraram a resolução da estenose e a normalização dos parâmetros de fluxo Doppler. O paciente apresentou resolução completa dos sintomas e permaneceu assintomático, após um ano de acompanhamento.

**Conclusões:**

A liberação robótica do ligamento arqueado mediano é uma opção minimamente invasiva segura e eficaz, proporcionando excelente visualização e dissecção precisa em uma região anatômica complexa. Essa técnica deve ser considerada uma abordagem valiosa para pacientes selecionados, com síndrome do ligamento arqueado mediano.

## INTRODUCTION

 Median arcuate ligament syndrome (MALS), also known as Dunbar syndrome, is an uncommon condition characterized by chronic abdominal pain resulting from external compression of the celiac trunk by the median arcuate ligament. The pathophysiology remains controversial and likely multifactorial, involving both vascular compression and neurogenic mechanisms related to irritation of the celiac plexus. Clinical presentation is often nonspecific, with postprandial pain and weight loss being the most frequently reported symptoms^7,14,17^. 

 Generally, the diagnosis is by exclusion due to the nonspecific symptoms that overlap with other forms of chronic intestinal ischemia, and relies on imaging studies, particularly computed tomography (CT) angiography (most sensitive and specific), which may demonstrate focal proximal stenosis of the celiac trunk with a characteristic hooked appearance, often accentuated during expiration. Duplex ultrasound may provide complementary hemodynamic information, although diagnostic criteria remain heterogeneous^10,11,16^. 

 The treatment consists of surgical decompression of the celiac trunk. The treatment is not always effective, and symptoms may persist, sometimes requiring revascularization of the celiac artery (endovascular with stents)^1,3,4,8,15^. 

 Minimally invasive approaches have increasingly replaced open techniques^2,5,6,12^. The robotic platform offers potential advantages in terms of three-dimensional visualization, ergonomic precision, and fine dissection in the retroperitoneal space^9,13,18^. 

 This report presents the technical aspects and imaging outcomes of robotic median arcuate ligament release using a case-based approach. 

## CASE PRESENTATION

 An 82-year-old man with a history of hypertension and dyslipidemia presented with severe epigastric pain exacerbated by meals and associated with progressive weight loss. Physical examination was unremarkable. 

**Figure 1:**
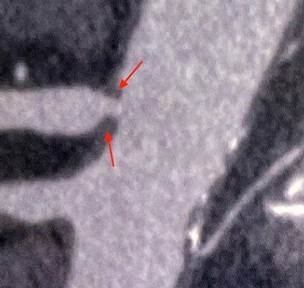
Computed tomography angiography demonstrates severe stenosis at the origin of the celiac trunk (red arrows), characterized by tapered contrast opacification.

 CT angiography demonstrated severe focal stenosis at the origin of the celiac trunk due to extrinsic compression, with restoration of normal caliber distally, consistent with median arcuate ligament syndrome (Figure 1). Based on clinical and imaging findings, surgical treatment was indicated. 

### Surgical technique

 The procedure was performed under general anesthesia using the da Vinci X robotic platform. After port placement for upper abdominal access and docking, extensive adhesiolysis was required due to prior abdominal surgeries. The hepatogastric ligament was divided to allow adequate exposure of the celiac trunk. 

 Lymphadenectomy around the celiac axis was performed to improve visualization. The median arcuate ligament was identified and carefully divided until full exposure of the anterior surface of the aorta was achieved. Fibrous and neural tissues surrounding the celiac trunk were meticulously dissected to ensure complete decompression. Total operative time was approximately 180 minutes, with no significant bleeding or intraoperative complications (Video 1 – https://youtu.be/WLmRu_5V9vs). 

### Postoperative outcome

 The patient was discharged on postoperative day five, with adequate pain control achieved using non-opioid analgesics on an as-needed basis. All preoperative symptoms resolved completely. 

**Figure 2:**
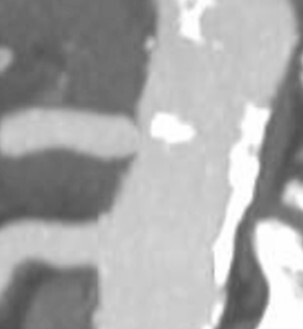
Sagittal view showing opacification of nearly the entire circumference of the celiac trunk, with only minimal angulation at its origin.

**Figure 3:**
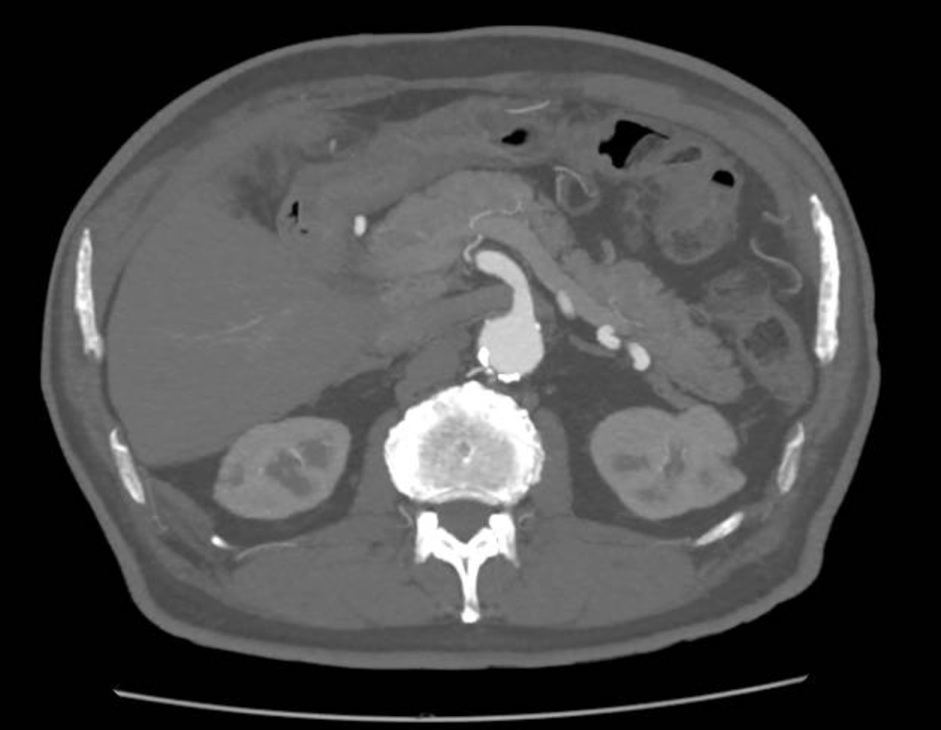
The minimal angulation seen on the sagittal view is not evident on the axial view.

**Figure 4:**
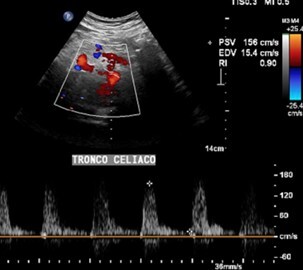
Color Doppler ultrasound shows a patent celiac trunk without hemodynamically significant stenosis, with low-resistance flow and normal velocities (155 cm/s; aortic ratio, 1.9).

 A follow-up CT angiography performed 60 days postoperatively demonstrated resolution of the celiac trunk stenosis (Figures 2 and 3). Color Doppler ultrasound showed a patent celiac trunk without evidence of stenosis, normal flow velocities (155 cm/s), low-resistance flow, and an aortic ratio of 1.9, with no significant respiratory-related velocity changes (Figure 4). At one year of follow-up, the patient remained asymptomatic. The patient signed the informed consent for this publication. 

## DISCUSSION

**Figure 5:**
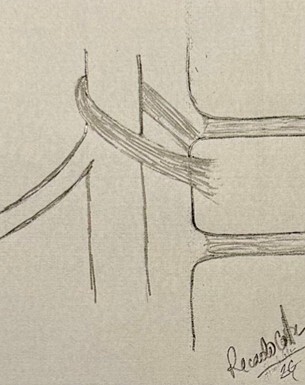
Author-drawn schematic illustration demonstrating normal insertion of the median arcuate ligament, with no extrinsic compression of the celiac trunk.

**Figure 6:**
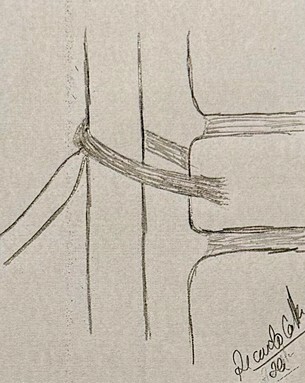
Author-drawn schematic illustration representing an anomalously low insertion of the median arcuate ligament causing extrinsic compression of the celiac trunk.

 MALS, also known as Dunbar syndrome, is a rare and often underdiagnosed condition caused by extrinsic compression of the celiac trunk by an anomalous low insertion of the median arcuate ligament (Figures 5 and 6). Although it predominantly affects women between the fourth and sixth decades of life, a clinically significant disease may also occur in elderly male patients when symptoms and imaging findings are concordant^3,7,17^. 

 The underlying mechanism of pain remains controversial. While some authors attribute symptoms to ischemia caused by reduced blood flow through the compressed celiac axis, others emphasize direct neural compression of the celiac ganglia. It is likely that both mechanisms coexist, supporting the need for complete vascular and neural decompression^6,8,14,17^. 

 The symptoms related to this syndrome include the triad of postprandial abdominal pain (due to visceral ischemia caused by compression), weight loss, and abdominal bruit, which may be associated with vomiting, diarrhea, and nausea^1,9^. The diagnosis is difficult and generally one of exclusion, due to nonspecific symptoms that overlap with other forms of chronic intestinal ischemia^3,14,17^. Confirmation occurs through imaging tests (CT or magnetic resonance imaging – MRI), and vascular tests, arteriography or Doppler ultrasound^10,11^. 

 The best imaging exam is angiotomography (Angio-CT), especially during expiration, which presents high precision for identifying celiac trunk stenosis due to compression, with good visualization of the underlying median arcuate ligament and adherent tissue, using three-dimensional image reconstruction^11,16^. 

 CT angiography allows for differential diagnosis with celiac trunk calcification, which can cause symptoms similar to Dunbar syndrome^2,11^. The treatment consists of decompressing the celiac artery through an open or minimally invasive (laparoscopic or robotic) approach. In some patients, revascularization of the celiac artery (open or endovascular) may be necessary due to the persistence of symptoms. 

 Surgical decompression remains the most effective treatment. Minimally invasive approaches have largely replaced open surgery for MALS, as they are associated with reduced postoperative pain, shorter hospital stays, and faster functional recovery^5,6,8^. The robotic platform further enhances these benefits by offering stable three-dimensional visualization, superior ergonomics, and precise dissection of fibrotic and neural tissues in a confined and anatomically complex region. Despite higher costs, available series demonstrate high rates of symptom resolution and low morbidity, supporting robotic release as a safe and effective option^3,9,13,18^. According to a study, 92% of patients who underwent surgical treatment for the resolution of Dunbar Syndrome via robotics showed complete improvement of symptoms and no recurrence. These findings further support robotic surgical treatment as a safe and effective approach^13^. 

 Published robotic series remain limited, with the largest cohort reporting approximately 27 patients^13^. Despite the small number of reported cases, robotic median arcuate ligament release has demonstrated favorable clinical outcomes and low complication rates^14^. This case reinforces existing evidence by illustrating complete symptom resolution and objective post-operative imaging normalization. The main limitation of this report is its single-patient design. However, given the rarity of MALS and the limited number of published robotic series, detailed technical reports with objective imaging correlation remain valuable to refine diagnostic and therapeutic strategies. 

## CONCLUSIONS

 Robotic median arcuate ligament release using the da Vinci platform is a safe, feasible, and effective minimally invasive approach for the treatment of MALS. The technique allows precise decompression of the celiac trunk, with excellent postoperative anatomical and hemodynamic outcomes. Robotic surgery should be considered a valuable option in selected patients. 

## Data Availability

The datasets generated and/or analyzed during the current study are available from the corresponding author upon reasonable request.
